# Preoperative neutrophil–to–lymphocyte ratio after chemoradiotherapy for esophageal squamous cell carcinoma associates with postoperative pulmonary complications following radical esophagectomy

**DOI:** 10.1186/s13741-024-00431-6

**Published:** 2024-07-02

**Authors:** Chien-Ming Lo, Hung-I. Lu, Yu-Ming Wang, Yen-Hao Chen, Yu Chen, Li-Chun Chen, Shau-Hsuan Li

**Affiliations:** 1 Department of Thoracic & Cardiovascular Surgery, Kaohsiung Chang Gung Memorial Hospital and Chang Gung University College of Medicine, Kaohsiung, Taiwan; 2 Deaprtment of Radiation Oncology, Kaohsiung Chang Gung Memorial Hospital and Chang Gung University College of Medicine, Kaohsiung, Taiwan; 3 Department of Hematology-Oncology, Kaohsiung Chang Gung Memorial Hospital and Chang Gung University College of Medicine, Kaohsiung, Taiwan; 4 No.123, Dapi Rd., Niaosong Dist., Kaohsiung City, 833 Taiwan

**Keywords:** Esophageal cancer, Neutrophil-to-lymphocyte ratio, Esophagectomy, Pulmonary complications

## Abstract

**Objectives:**

Esophagectomy after chemoradiotherapy is associated with an increased risk of surgical complications. The significance of preoperative neutrophil-to-lymphocyte ratio and platelet-to-lymphocyte ratio after chemoradiotherapy in predicting pulmonary complications following radical esophagectomy in esophageal squamous cell carcinoma patients receiving preoperative chemoradiotherapy remains unknown. We aimed to investigate the utility of neutrophil-to-lymphocyte ratio and platelet-to-lymphocyte ratio in predicting the pulmonary complications of esophagectomy after preoperative chemoradiotherapy.

**Methods:**

We retrospectively reviewed 111 consecutive patients with stage III esophageal squamous cell carcinoma who received preoperative chemoradiotherapy followed by esophagectomy between January 2009 and December 2017. Laboratory data were collected before the operation and surgical outcomes and complications were recorded. We calculated neutrophil-to-lymphocyte ratio and platelet-to-lymphocyte ratio and correlated them with the clinical parameters, postoperative complications, overall survival, and disease-free survival.

**Results:**

Postoperative complications were observed in 75 (68%) patients, including 32 (29%) with pulmonary complications. The preoperative neutrophil-to-lymphocyte ratio of ≥ 3 (*P* = 0.008), clinical T4 classification (*P* = 0.007), and advanced stage IIIC (*P* = 0.012) were significantly associated with pulmonary complications. Pulmonary complication rates were 15% and 38% in patients with preoperative neutrophil-to-lymphocyte ratio of < 3 and ≥ 3, respectively. Preoperative neutrophil-to-lymphocyte ratio was not associated with the oncological stratification such as pathological T classification, pathological N classification, and pathological AJCC stage. The 3-year overall survival rates were 70% and 34% in patients with preoperative neutrophil-to-lymphocyte ratio of < 3 and ≥ 3, respectively (*P* = 0.0026). The 3-year disease-free survival rates were 57% and 29% in patients with preoperative neutrophil-to-lymphocyte ratio of < 3 and ≥ 3, respectively (*P* = 0.0055). The preoperative neutrophil-to-lymphocyte ratio of ≥ 3 was independently associated with more pulmonary complications, inferior overall survival, and worse disease-free survival.

**Conclusions:**

Elevated preoperative neutrophil-to-lymphocyte ratio after chemoradiotherapy is independently associated with higher pulmonary complication rate following radical esophagectomy and poor prognosis in patients with esophageal squamous cell carcinoma receiving preoperative chemoradiotherapy. Preoperative neutrophil-to-lymphocyte ratio is routinely available in clinical practice and our findings suggest it can be used as a predictor for pulmonary complications after esophagectomy in patients with esophageal squamous cell carcinoma receiving preoperative chemoradiotherapy.

## Introduction

Esophageal cancer has a poor prognosis despite several advances in diagnosis and treatment. Preoperative chemoradiotherapy followed by esophagectomy is a major treatment modality for patients with locally advanced ESCC. After chemoradiotherapy, 20%–50% of patients with locally advanced ESCC can achieve a pathological complete response(Lo et al. 2021). Esophagectomy is one of the main treatment modalities for esophageal cancer; however, it results in high surgical morbidity and mortality, especially in patients who received chemoradiotherapy and underwent esophageal squamous cell carcinoma (ESCC) histology (Blencowe et al. 2012; Steyerberg et al. 2006). Even with the recent advances in minimally invasive esophagectomy, patients who undergo chemoradiotherapy before esophagectomy have a high rate of postoperative complications, especially pulmonary complications, which is one of the leading causes of postoperative mortality (Kitagawa and Matsuda 2020; Low et al. 2015). Fujita et al. (Fujita et al. 2005) reported that six (40%) of 15 patients with cT4N0-1M0 ESCC receiving 36 Gy preoperative chemoradiotherapy had pulmonary complications after esophagectomy, including five cases of pneumonia and one of acute respiratory distress syndrome (ARDS), and 7% postoperative death. Burmeister et al. (Burmeister et al. 2005) reported a 20% pulmonary complication rate and 5% surgery-related deaths in 105 patients with cT1-3N0-1M0 esophageal cancer receiving 35 Gy preoperative chemoradiotherapy. Stahl and others (Stahl et al. 2005) reported that seven (11.3%) of 62 patients with cT3-4N0-1M0 ESCC receiving 40 Gy preoperative chemoradiotherapy suffered from postoperative death, and 70% of the patients developed at least one severe complication. Tepper et al. (Tepper et al. 2008) reported a 38% pulmonary complication rate in 24 patients with cT1-3NxM0 esophageal cancer receiving 50.4 Gy preoperative chemoradiotherapy. Hagen et al. (Hagen et al. 2012) reported that 78 (48%) of 168 patients with cT1N1M0 or T2-3N0-1M0 esophageal cancer receiving 41.4 Gy preoperative chemoradiotherapy had pulmonary complications and 4% hospital mortality. High surgical morbidity worsens the overall survival (OS) and causes concurrent chemoradiotherapy in patients; therefore, it is imperative and valuable to identify patients who are at high risk for surgical complications of esophagectomy after chemoradiotherapy. Identifying the predictor before surgery could help us adjust the treatment policy such as avoiding high-risk surgery, choosing better timing for surgery, close follow-up after surgery, or proper handling in the intensive care unit, which could decrease the complication rate of esophagectomy and improving the treatment outcome.

Systemic inflammatory responses commonly develop in cancer patients. Many previous studies have also shown that the systemic inflammatory response has a large impact on treatment prognosis of many cancers (Corbeau et al. 2020; Almasaudi et al. 2020). Recently, the neutrophil-to-lymphocyte ratio (NLR) and platelet-to-lymphocyte ratio (PLR) have been recognized as potential markers for systemic inflammatory response, and a higher NLR or PLR have been reported to be associated with poor treatment outcomes in a variety of cancers (Corbeau et al. 2020; Almasaudi et al. 2020). Previous studies (Koh et al. 2021; Wang et al. 2021; Wu et al. 2021; Gao et al. 2019; Chen et al. 2019; Anand et al. 2021; Tustumi et al. 2020; Hyder et al. 2016) have reported that elevated NLR or PLR predicted poor survival in patients with esophageal cancer receiving different treatment modalities such as esophagectomy, preoperative chemoradiotherapy followed by esophagectomy, or definitive chemoradiotherapy. Clinically, the NLR and PLR are quick, routinely available, and easily measurable markers (Zahorec 2001). In addition to predicting prognosis of cancer patients, postoperative NLR or PLR has also been used as a predictor of surgical complications in patients with gastric cancer, pancreatic cancer, and non-small cell lung cancer undergoing surgery (Ortiz-Lopez et al. 2021; Bora Makal and Yildirim 2020; Wang et al. 2020; Ida et al. 2019; Mungan et al. 2020). For patients with esophageal cancer who underwent esophagectomy, Vulliamy et al. (Vulliamy et al. 2016) reported that postoperative elevated NLR predicted complications of Ivor-Lewis esophagectomy; however, the NLR and PLR were postoperative, not preoperative. Moreover, patients with esophageal cancer in a study by Vulliamy et al. (Vulliamy et al. 2016) did not receive chemoradiotherapy before esophagectomy, and most of them had esophageal adenocarcinoma, not ESCC.

To the best of our knowledge, the significance of preoperative NLR and PLR after chemoradiotherapy in predicting pulmonary complications of esophagectomy in patients with ESCC receiving preoperative chemoradiotherapy remains unclear. We investigated preoperative NLR and PLR after chemoradiotherapy instead of postoperative NLR and PLR because the systemic inflammatory response, which is represented by NLR and NLR, may be induced to varying degrees by chemoradiotherapy before esophagectomy in patients with ESCC, which may further affect the surgical complications of esophagectomy. Therefore, this study aims to evaluate the utility of preoperative NLR and PLR after chemoradiotherapy in predicting pulmonary complications of esophagectomy after preoperative chemoradiotherapy.

## Patients and Methods

### Patient population

Patients with clinical stage III ESCC who underwent preoperative chemoradiotherapy followed by esophagectomy between January 2009 and December 2017 at Kaohsiung Chang Gung Memorial Hospital were reviewed retrospectively. Cancer staging was based on the 7th American Joint Committee on Cancer (AJCC) staging system. The inclusion criteria of our study were as follows: (1) pathologically confirmed diagnosis of ESCC excluding other histological types; (2) patients who underwent preoperative chemoradiotherapy followed by esophagectomy; (3) patients with preoperative complete blood count (CBC) and differential count (DC) results 3 days before esophagectomy; (4) and AJCC clinical stage III. The exclusion criteria of this study were as follows: (1) Induction treatment with chemotherapy or radiotherapy alone; (2) cervical location of esophageal cancer; (3) patients with synchronous cancer such as head and neck cancer; (4) progressive disease with distant metastases during chemoradiotherapy; and (5) the inability to evaluate response to chemoradiotherapy or to receive esophagectomy. During this period, 1,131 patients with esophageal cancer received treatment at our hospital, and 111 patients diagnosed with 7th AJCC clinical stage III ESCC receiving preoperative chemoradiotherapy followed by esophagectomy were identified.

In our study, patients were evaluated by a multidisciplinary team, including a thoracic surgeon, medical oncologist, radiation oncologist, radiologist, and gastroenterologist. Pretreatment staging evaluation included physical examination, endoscopy, contrast-enhanced computed tomography (CT) scans from the neck to the upper abdomen, positron emission tomography-computed tomography (PET-CT) scans, and endoscopic ultrasound. Tumor node metastasis stage was determined according to the 7th AJCC staging system (Edge C.C., Fritz AG, Greene FL, Trotti A, C.C., Fritz AG, Greene FL, Trotti A 2010). The study was approved by the Institutional Review Board of Chang Gung Memorial Hospital.

### Preoperative chemoradiotherapy protocol

For patients receiving preoperative chemoradiotherapy, we administered two cycles of concurrent cisplatin and 5-fluorouracil-based chemotherapy and radiotherapy. Chemotherapy consisted of cisplatin (75 mg/m^2^; 4-h drip) on day 1 and 5-fluorouracil (1000 mg/m^2^; continuous infusion) on days 1–4 every 4 weeks. Radiotherapy was delivered in five daily fractions per week. Three-dimensional conformal radiotherapy via a four-field technique or intensity-modulated radiotherapy with 6 MV or 10-MV photons was used. The gross target volume was defined as the gross tumor and gross lymph nodes on CT and/or PET/CT images. The clinical target volume (CTV) comprehensively covered the esophagus, mediastinal lymph nodes, bilateral neck, and supraclavicular lymph nodes. The planning target volume (PTV) was expanded from the CTVs with a 0.5–1.0 cm margin in all directions. The total radiotherapy dose to the PTV was 50–50.4 Gy in 25–28 fractions administered 5 days per week. Within 3–4 weeks following the end of irradiation, CT from the neck to the upper abdomen, endoscopy, and PET-CT were performed to observe the treatment response. The multidisciplinary team then reviewed the clinical information to determine if the lesions were resectable. If lesions were classified as resectable and patients were medically fit for esophagectomy, surgery was advised 6–12 weeks after the end of chemoradiotherapy. Pathological complete response was defined as the complete disappearance of all viable cancer cells in all surgical specimens, including the primary esophageal tumor and lymph nodes.

### Neutrophil-to-lymphocyte ratio (NLR) and platelet-to-lymphocyte ratio (PLR)

All patients underwent complete blood count (CBC) and differential count (DC) within 3 days before esophagectomy. NLR was defined as the ratio of the neutrophil count to the lymphocyte count. PLR was defined as the ratio of the platelet count by the lymphocyte count. The cut-off levels of NLR and PLR for predicting pulmonary complications of esophagectomy after chemoradiotherapy were determined using receiver operating characteristic analyses.

### Surgery and complications

We had two well-trained thoracic surgeons in the team who performed approximately 40–60 esophagectomies per year. The patients were familiar with minimally invasive esophagectomy. All patients received double-lumen endotracheal tube intubation for one-lung ventilation. Esophagectomy with two-field lymphadenectomy was performed using thoracoscopy-assisted and then laparoscopic-assisted gastric tube harvesting for esophageal reconstruction without thoracic duct ligation. Cervical esophagogastrostomy was performed in a hand-sew fashion. Patients were transferred to the intensive care unit for critical care after the procedure and were transferred to the ordinary ward after extubation.

All postoperative complications such as pneumonia, ARDS, empyema, pneumothorax, anastomosis leak, vocal cord paralysis, wound infection, sepsis, etc. were recorded during the first 30 days postoperatively and graded according to the Clavien-Dindo classification (Dindo et al. 2004). Complications of the lungs, such as pneumonia, acute respiratory distress syndrome (ARDS), empyema, and pneumothorax, were classified as pulmonary complication Each postoperative complication was categorized to the international consensus (Low et al. 2015). That is, the pulmonary complications included pneumonia, pleural effusion requiring additional drainage procedure,

pneumothorax, acute respiratory distress syndrome, and tracheobronchial injury. Anastomosis leak belonged to gastrointestinal complications. We confirmed the vocal cord palsy by clinical hoarseness symptom and consulted the ENT surgeon to survey vocal cord palsy under nasopharyngeal scope at the same time.

### Follow-up, overall survival, and disease-free survival

Post-treatment follow-ups were performed every 3 months in 1 and 2 years, every 6 months in 3–5 years, and after 1 year thereafter. Disease-free survival (DFS) was defined as the date of esophagectomy to the date of recurrence or death from any cause without evidence of recurrence. OS was defined as the time between the date of diagnosis to the date of death.

### Statistical analyses

Statistical analyses were performed using the Statistical Package for the Social Sciences (SPSS, ver. 25, Chicago, IL, USA). A χ^2^ test was used to compare data between the two groups. We also performed multiple variance analyses for pulmonary complications and all complications using logistic regression. For survival outcomes, the Kaplan–Meier method was used for univariate analysis, and the difference between survival curves was analyzed using the log-rank test. For multivariate survival analyses, all variables were entered into the Cox regression model in a stepwise forward fashion to analyze their relative prognostic importance. For all analyses, two-sided tests of significance were used with a P-value of < 0.05 considered statistically significant.

## Results

### Patient characteristics and postoperative complications

The clinicopathologic features of the 111 study patients (108 men and three women) are listed in Table 1. We compared age, clinical stage, T classification, N classification, tumor location, tumor grade and pathologic complete response.

**Table 1:** Clinicopathologic features of 111 patients with stage III esophageal squamous cell carcinoma receiving preoperative chemoradiotherapy followed by esophagectomy

Parameters	No. of cases (%)
Age (years) (mean: 52.69, median: 52, range: 36–77)	
Clinical 7th AJCC stage	
IIIA	24 (22%)
IIIB	17 (15%)
IIIC	70 (63%)
Clinical T classification	
T2	3 (3%)
T3	47 (42%)
T4a	8 (7%)
T4b	53 (48%)
Clinical N classification	
N0	4 (4%)
N1	47 (42%)
N2	42 (38%)
N3	18 (16%)
Tumor grade	
Grade 1	15 (14%)
Grade 2	71 (64.0%)
Grade 3	25 (23%)
Primary tumor location	
Upper	36 (33%)
Middle	48 (43%)
Lower	27 (24%)
Pathological complete response	
Absent	73 (66%)
Present	38 (34%)

*AJCC* American Joint Committee on Cancer

Postoperative complications and Clavien-Dindo classification are listed in Table 2. There were 29% pulmonary complications and 68% all complications. The detail information about complication listed in Table 2.

**Table 2:** Postoperative complications and mortality after esophagectomy in 111 patients with stage III esophageal squamous cell carcinoma receiving preoperative chemoradiotherapy

Complications	No. of cases (%)	Clavien-Dindo classification grade (n.)
Pulmonary complications	32 (29%)	
Pneumonia	25 (22%)	V (6);IVa(7);IIIa(2);II(5);I (5)
Acute respiratory distress syndrome	13 (12%)	V(8);IVa(5)
Pleural effusion requiring additional drainage procedure	5 (5%)	IVa(1);IIIb(1);IIIa(3)
Pneumothorax	4 (4%)	IIIa(1);I(3)
Tracheobronchial injury	1 (1%)	IIIb(1)
Gastrointestinal Anastomosis leak	21 (19%)	V(2);IVa(2);IIIa(9);IIIb(1);II(7)
Neurologic/Psychiatric Vocal cord paralysis	15 (14%)	II(11);I(4)
Infection Sepsis	3 (3%)	V(1);IVa(1);IIIa(1)
Other Complications Chylothorax	5 (5%)	IVa(2);IIIb(2);I(1)
All complications	75 (68%)	V(12);IVa(8);IIIa(20);IIIb(5);II(18);I (12);0(36)
30-day mortality	3 (3%)	
Hospital mortality	12 (10%)	

### Correlations of clinicopathologic parameters with neutrophil-to-lymphocyte ratio and platelet-to-lymphocyte ratio

The correlations of clinicopathologic parameters with NLR and PLR are summarized in Table 3. We did not find any significant correlation between NLR or PLR and age, primary tumor location, clinical T classification, clinical N classification, clinical 7th AJCC stage, tumor grade, pathological complete response, pathological T classification, pathological N classification, pathological AJCC stage, duration from the end of chemoradiotherapy to surgery, surgery time and blood loss in operation.

**Table 3:** Associations of clinicopathological parameters with NLR and PLR in 111 patients with stage III esophageal squamous cell carcinoma receiving preoperative chemoradiotherapy followed by esophagectomy

Parameters		NLR			PLR		
		< 3	≥ 3	*P*-value	< 140	≥ 140	*P*-value
Age, years	< 52	20	31	0.66	12	39	0.09
	≥ 52	26	34		23	37	
Primary tumor location	Upper/Middle	31	44	0.97	23	52	0.78
	Lower	15	21		12	24	
Primary tumor location	Upper	11	15	0.92	6	20	0.29
	Middle/Lower	35	50		29	56	
Clinical T classification	T1/2/3	22	28	0.62	17	33	0.61
	T4	24	37		18	43	
Clinical N classification	N0/1	23	28	0.47	18	33	0.43
	N2/3	23	37		17	43	
Clinical 7th AJCC stage	IIIA/IIIB	18	23	0.69	15	26	0.38
	IIIC	28	42		20	50	
Histological grading	Grade 1/2	35	51	0.77	27	59	0.95
	Grade 3	11	14		8	17	
Pathological CR	Absent	28	45	0.36	25	48	0.39
	Present	18	20		10	28	
Duration (end of CRT to lab data) (days)		72.6	73.4	0.54	67.4	67.8	0.87
Blood Loss (ml)		273	259	0.50	301	248	0.36
Surgery Time (hrs)		9.82	9.89	0.71	10	9.9	0.79
Pathological T classification	CR	20	23	0.17	10	33	0.07
	1	9	4		9	4	
	2	6	6		6	6	
	3	7	20		6	21	
	4	4	12		4	12	
Pathological N classification	0	35	44	0.35	27	52	0.12
	1	9	18		5	22	
	2	0	2		1	1	
	3	2	0		2	0	
Pathological Stage	CR	18	20	0.35	10	28	0.35
	1	5	2		5	2	
	2	15	19		13	21	
	3	8	22		7	23	
	4	0	1		0	1	

*AJCC* American Joint Committee on Cancer, *NLR* neutrophil-to-lymphocyte ratio, *PLR* platelet-to-lymphocyte ratio, *CR* complete response, *CRT* chemoradiotherapy

### Correlation between postoperative complications and clinicopathologic parameters, neutrophil-to-lymphocyte ratio, and platelet-to-lymphocyte ratio

The correlation between postoperative complications and clinicopathological parameters, NLR, and PLR is presented in Table 4. NLR ≥ 3 was significantly associated with a higher postoperative complication rate (*P* = 0.012). Clinical T classification T4 (*P* = 0.007), clinical 7th AJCC stage IIIC (*P* = 0.012), and NLR ≥ 3 (*P* = 0.008) were significantly associated with more pulmonary complications.

**Table 4:** Associations between clinicopathological parameters and surgical complications in 111 patients with stage III esophageal squamous cell carcinoma receiving preoperative chemoradiotherapy followed by esophagectomy

Parameters		All complications			Pulmonary complications		
		Absent	Present	*P*-value	Absent	Present	*P*-value
Age, years	< 52	13	38	0.15	38	13	0.47
	≥ 52	23	37		41	19	
Primary tumor location	Upper/Middle	23	52	0.57	51	24	0.29
	Lower	13	23		28	8	
Primary tumor location	Upper	6	20	0.24	17	9	0.46
	Middle/Lower	30	55		62	23	
Clinical T classification	T2/3	16	34	0.93	42	8	0.007*
	T4	20	41		37	24	
Clinical N classification	N0/1	18	33	0.55	39	12	0.26
	N2/3	18	42		40	20	
Clinical 7th AJCC stage	IIIA/IIIB	14	27	0.77	35	6	0.012*
	IIIC	22	48		44	26	
Histological grading	Grade 1/2	29	57	0.59	63	23	0.37
	Grade 3	7	18		16	9	
Pathological CR	Absent	20	53	0.12	50	23	0.39
	Present	16	22		29	9	
NLR	< 3	21	25	0.012*	39	7	0.008*
	≥ 3	15	50		40	25	
PLR	< 140	12	23	0.78	28	7	0.16
	≥ 140	24	52		51	25	

*AJCC* American Joint Committee on Cancer, *NLR* neutrophil-to-lymphocyte ratio, *PLR* platelet-to-lymphocyte ratio, *CR* complete response

^*^Statistically significant

We then performed multivariate logistic regression to determine the impact of different clinicopathologic parameters on all complications and pulmonary complications. We found that NLR ≥ 3 (*P* = 0.014, hazard ratio (HR): 2.800, 95% confidence interval (CI): 1.235–6.346) was independently and significantly associated with all complications. The surgical complication rates were 54% and 77% in patients with NLR < 3 and NLR ≥ 3, respectively. Additionally, further analyses showed that NLR ≥ 3 (*P* = 0.011; HR: 3.564; 95% CI: 1.344–9.456) and clinical T classification (T4, *P* = 0.009, HR: 3.484, 95% CI: 1.358–8.939) were independently and significantly associated with pulmonary complications. The pulmonary complication rates were 15% and 38% in patients with NLR < 3 and NLR ≥ 3, respectively.

### Survival analyses

**Fig. 1:**
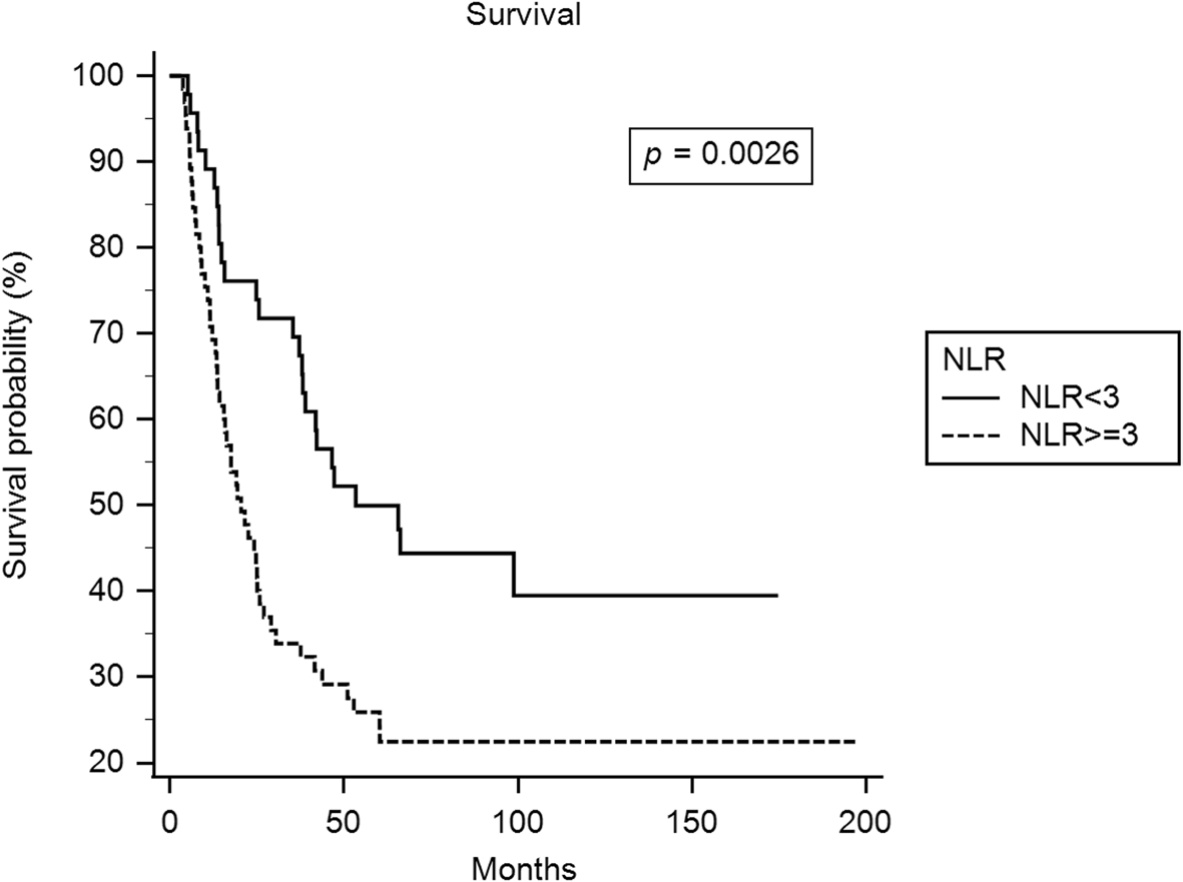
Neutrophil-to-lymphocyte ratio (NLR) ≥ 3 is significantly associated with worse overall survival (**A**) and inferior disease-free survival (**B**) in patients with locally advanced esophageal squamous cell carcinoma receiving preoperative chemoradiotherapy followed by esophagectomy (solid line indicates NLR < 3, dotted line indicates NLR ≥ 3)

The correlations of clinicopathologic parameters with OS and DFS are summarized in Table 5. The univariate survival analyses revealed that clinical T classification (T4, P = 0.011), clinical 7th AJCC stage IIIC (*P* = 0.039), NLR ≥ 3 (*P* = 0.0026, Fig. 1A), absence of pathological complete response (P = 0.0018), and pulmonary complications (*P* < 0.0001) were significantly associated with worse OS. Additionally, clinical T classification (T4, *P* = 0.01), clinical 7th AJCC stage IIIC (*P* = 0.04), NLR ≥ 3 (*P* = 0.0055, Fig. 1B), absence of pathological complete response (*P* = 0.0011), and pulmonary complications (*P* < 0.0001) were significantly associated with inferior DFS. Using the multivariate analysis with Cox proportional-hazards regression model, we found that clinical T classification (T4, *P* = 0.006, HR: 1.948, 95% CI: 1.216–3.121), NLR ≥ 3 (*P* = 0.003, HR: 2.068, 95% CI: 1.275–3.353), and absence of pathological complete response (*P* = 0.001, HR: 2.514, 95% CI: 1.465–4.314) were independent and significant prognosticators for worse OS. Additionally, clinical T classification (T4, *P* = 0.008, HR: 1.904, 95% CI: 1.187–3.054), NLR ≥ 3 (*P* = 0.009, HR: 1.899, 95% CI: 1.172–3.077), and absence of pathological complete response (*P* = 0.001, HR: 2.536, 95% CI: 1.474–4.361) were also independent and significant prognostic factors for poor DFS. The 3-year OS rates were 70% and 34% in patients with NLR < 3 and NLR ≥ 3, respectively. The 3-year DFS rates were 57% and 29% in patients with NLR < 3 and NLR ≥ 3, respectively.

**Table 5:** Results of univariate log-rank analysis of prognostic factors for overall survival and disease-free survival in 111 patients with stage III esophageal squamous cell carcinoma receiving preoperative chemoradiotherapy followed by esophagectomy

Factors	No. of patients	Overall survival (OS)		Disease-free survival (DFS)	
		3-year OS rate (%)	P-value	3-year DFS rate (%)	*P*-value
Age, years					
< 52	51	49	0.88	41	0.96
≥ 52	60	48		40	
Clinical 7th AJCC stage					
IIIA/IIIB	41	66	0.039*	54	0.04*
IIIC	70	39		33	
Clinical T classification					
T2/3	50	66	0.011*	54	0.01*
T4	61	34		30	
Clinical N classification					
N0/1	51	53	0.87	39	0.97
N2/3	60	45		42	
Tumor grade					
Grade 1/2	86	51	0.26	41	0.30
Grade 3	25	40		40	
Primary tumor location					
Upper	26	46	0.59	42	0.69
Middle/Lower	85	49		40	
Primary tumor location					
Upper/Middle	75	48	0.34	43	0.29
Lower	36	50		36	
NLR					
< 3	46	70	0.0026*	57	0.0055*
≥ 3	65	34		29	
PLR					
< 140	35	63	0.396	46	0.53
≥ 140	76	42		38	
Pathological CR					
Absent	73	40	0.0018*	27	0.0011*
Present	38	69		66	
Surgical complications					
Absent	36	55	0.12	47	0.13
Present	75	44		36	
Pulmonary complications					
Absent	79	59	< 0.0001*	49	< 0.0001*
Present	32	20		17	

*NLR* neutrophil-to-lymphocyte ratio, *PLR* platelet-to- lymphocyte ratio, *CR* complete response

^*^Statistically significant

## Discussion

Patients with esophageal cancer have a poor prognosis owing to delayed diagnosis, and most patients are diagnosed at an advanced stage. Preoperative chemoradiotherapy followed by esophagectomy is a major treatment modality for patients with locally advanced ESCC. After chemoradiotherapy, 20%–50% of patients with locally advanced ESCC can achieve a pathological complete response (Lo et al. 2021). However, chemoradiotherapy may increase postoperative complications, especially pulmonary complications during esophagectomy. If surgical complications caused mortality, then the effort and effect of preoperative chemoradiotherapy were in vain. Thus, we tried to identify the predictor to inform surgeons about patients at high risk for postoperative complications of esophagectomy who may defer surgery to prevent or reduce the risk of complications or need intensive care postoperatively. In our study, we found that patients with higher preoperative NLR had significantly more pulmonary complications after esophagectomy and chemotherapy. We suggest that surgeons should assess NLR to gain some prior information of the risks involved in the surgery.

NLR has shown its privilege in oncology for stratification of cancer, correlates with the tumor size, stage of tumors, metastatic potential and lymphatic invasion. However, in our study, we found that preoperative NLR after chemoradiotherapy was not associated with the oncological stratification such as pathological T classification, pathological N classification, and pathological AJCC stage, indicating that preoperative NLR may be affected by factors other than tumor staging. We observed that high preoperative NLR was associated with more pulmonary complications of esophagectomy in patients with ESCC who received preoperative chemoradiotherapy. The reason why a high preoperative NLR after chemoradiotherapy was associated with more pulmonary complications may need further exploration. We suggested that the systemic inflammatory response especially radiation pneumonitis, which is represented by NLR, may be induced to varying degrees by chemoradiotherapy before esophagectomy in patients receiving preoperative chemoradiotherapy followed by esophagectomy. We suggest that some patients may develop asymptomatic radiation pneumonitis or radiation pneumonitis with only mild symptoms after chemoradiotherapy. However, it is difficult to detect radiation pneumonitis before esophagectomy because patients may be asymptomatic or have only mild symptoms. A previous study reported that grade 1 radiation pneumonitis did not show any clinical symptoms or signs (Kim et al. 2011). If these patients receive radical esophagectomy, postoperative pulmonary complications along with radiation pneumonitis after chemoradiotherapy may further worsen lung function, cause respiratory failure, and even mortality. Additionally, patients may not have symptom before esophagectomy because they may be in the latent stage of radiation pneumonitis. After esophagectomy, radiation pneumonitis may enter into acute stage which may further aggravate pulmonary complication of esophagectomy. The original Gr1 radiation pneumonitis may progress to Gr2, even Gr3 or Gr4 over time. Thus, preoperative NLR could provide some clues to early detection of high-risk groups of patients before surgery and remind surgeons that they can defer radical esophagectomy after radiation pneumonitis improves. Yang et al. (Yang et al. 2021) showed that NLR can predict radiation pneumonitis in 174 patients with esophageal cancer receiving radiotherapy or chemoradiotherapy. Lee et al. (Lee et al. 2018) reported that NLR could predict radiation pneumonitis in 61 patients with stage III non-small cell lung cancer receiving definite concurrent chemoradiotherapy. These findings further support our inferences.

Patients with locally advanced ESCC have a poor prognosis. Previous studies (Blencowe et al. 2012; Low et al. 2015; Vrba et al. 2019) have reported that a high rate of postoperative complications, especially pulmonary complications, is one of the reasons that lead to poor prognosis. In our study, we found that patients with postoperative pulmonary complications had worse OS and DFS, which further supports previous findings. If we could predict pulmonary complications before surgery, there is a possibility to improve OS in esophageal cancer patients. To the best of our knowledge, other studies that discuss NLR and esophageal cancer suggest that NLR could predict outcome of esophageal cancer (Yuan et al. 2014; Sherry et al. 2019; Li et al. 2019; Cai et al. 2020; Barbetta et al. 2018); however, the studies did not mention the prediction of esophagectomy complications after chemoradiotherapy. Vulliamy et al. (Vulliamy et al. 2016) showed that postoperative elevated NLR was correlated with complications of Ivor-Lewis esophagectomy. however, the NLR and PLR were postoperative, not preoperative. In our study, we found a relationship between preoperative NLR and pulmonary complication of esophagectomy after chemoradiotherapy. We investigated the significance of preoperative NLR and PLR instead of postoperative NLR and PLR because the systemic inflammatory response, which is represented by NLR and PLR, may be induced to varying degrees by chemoradiotherapy before esophagectomy in patients receiving preoperative chemoradiotherapy followed by esophagectomy, which may further affect the surgical complications of esophagectomy. The advantage of preoperative NLR over postoperative NLR is that surgeons may adjust the surgical schedule or prepare intensive postoperative care planning earlier, which may improve treatment outcomes in these patients. Furthermore, the NLR is easier to obtain before surgery, compared to other biomarkers. We can repeat the procedure for each patient before the surgery and took it into consideration in the preoperative survey. If the NLR is high, the patient may have higher risk to develop pulmonary complication after esophagectomy. We can postpone the surgery for several days with best supportive care to improve the inflammation status of patients and to improve their outcomes. Furthermore, there are also some perioperative managements which may prevent pulmonary complications and improve surgical outcomes including a single-lumen endotracheal tube intubation with artificial carbon dioxide pneumothorax in esophagectomy(Chuang et al. 2021; Nomura et al. 2020; Ninomiya et al. 2017), and fluid restriction(Low et al. 2007; Kita et al. 2002)..

Our study has some limitations. First, this was a retrospective study. Some predisposing factors may have influenced the results; however, we could not identify them comprehensively because of the retrospective design of our study. Second, different surgeon had different surgical skills that might have led to some bias in complications after surgery. For these reasons, we recommend further prospective studies to clarify our findings.

In conclusion, elevated preoperative neutrophil-to-lymphocyte ratio after chemoradiotherapy is independently associated with higher pulmonary complication rate following radical esophagectomy and poor prognosis in patients with esophageal squamous cell carcinoma receiving preoperative chemoradiotherapy. Our findings suggest that preoperative NLR after chemoradiotherapy could be used as a marker for patients with locally advanced ESCC receiving preoperative chemoradiotherapy followed by esophagectomy to predict the pulmonary complications of radical esophagectomy.

## Data Availability

All data generated or analyzed during this study are included in this published article.

## Abbreviations

ESCC: Esophageal squamous cell carcinoma
ARDS: Acute respiratory distress syndrome
OS: Overall survival
NLR: Neutrophil-to-lymphocyte ratio
PLR: Platelet-to-lymphocyte ratio
AJCC: American Joint Committee on Cancer
CBC: Complete blood count
DC: Differential count
CT: Computed tomography
PET-CT: Positron emission tomography-computed tomography
CTV: Clinical target volume
PTV: Planning target volume
HR: Hazard ratio
CI: Confidence interval
